# PW02-002 - Single MVK mutation and recurrent fevers

**DOI:** 10.1186/1546-0096-11-S1-A142

**Published:** 2013-11-08

**Authors:** K Barron, A Ombrello, D Goldsmith, I Aksentijevich, A Jones, D Kastner

**Affiliations:** 1 National Institutes of Health, Bethesda, PA, USA; 2 Drexel University Coll Med, PA, USA

## Introduction

HyperIgD syndrome is an autoinflammatory disorder caused by mutations in the *MVK* gene. While mutations in most patients follow autosomal recessive inheritance, we have identified a cohort of patients with recurrent fevers and only 1 mutation in the *MVK* gene.

## Objectives

To compare clinical features in those with 1 vs. 2 *MVK* mutations and to report therapeutic responses in all.

Patients were evaluated at the NIH. Clinical and laboratory information were collected at each visit.

## Methods

Patients were evaluated at the NIH. Clinical and laboratory information were collected at each visit.

## Results

31 pts with mutations in *MVK* were evaluated: 22 had 2 mutations (21 with V377I and 1 other mutation; 1 with V203A/H380R), 9 had only 1 mutation after testing the whole gene (8 with V377I, 1 with I268V). The carrier frequency of V377I in our control Caucasian population is 0.3% (2/739). In contrast, in 344 independent cases of recurrent fever submitted for *MVK* testing, 8 bore a single copy of V377I for a frequency of 2.3%.

Clinical or laboratory presentation at the time of a flare was compared between the 2 groups. There was no significant difference with regard to age of onset, duration of flares, frequency of flares, flares after immunizations, GI symptoms, oral ulcers, sore throat, arthralgia, or adenopathy associated with flares. Rash was more common in pts with 2 mutations, 20/22 compared to 4/9 in those with one mutation (p=.01). While there was no difference in level of IgG, IgA was increased in those with 2 mutations (452 +230 mg/dl) compared to those with 1 mutation (230 + 175) (p=.01), as well as level of IgD, (95 + 95, 2 mutations, vs. 8.3 + 7.4, 1 mutation, p=.01).

Since there was no significant difference in clinical presentation, other than presence of rash and levels of IgA and IgD, pts were considered together to evaluate their therapeutic responses. Of 8 pts treated with colchicine, 7 reported no response, 1 reported some improvement. Of 27 pts treated with prednisone at the time of a flare, 18 noted some improvement; 7 reported either none or shortening of the interval before next flare. Of 15 pts receiving montelukast, 4 reported some improvement; 11 reported none. Of 19 pts receiving intermittent anakinra at the time of a flare, 13 reported some improvement, 3 too early to assess efficacy, and 3 no improvement including one who developed acute renal failure. 5 pts received daily anakinra, with 4 reporting some improvement and 1 too early to assess. Of 9 pts receiving etanercept, 4 reported improvement, 5 report none.

## Conclusion

Aside for the presence of rash and higher IgA and IgD levels in those children with 2 *MVK* mutations, there are no significant clinical differences between these groups. There are no clear trends that allow identification or predictability of the disease course in children with either 1 or 2 mutations. Given the higher frequency of V377I heterozygotes in our patient cohort compared to the general population, our data suggest that under some circumstances this may be associated with recurrent fevers. Therapeutic options for children with *MVK* mutations include intermittent prednisone or anakinra, either given intermittently or daily; however, not all patients respond to therapy and there are associated adverse events in some patients.

## Disclosure of interest

None declared.

